# *Hox* genes and evolution

**DOI:** 10.12688/f1000research.7663.1

**Published:** 2016-05-10

**Authors:** Steven M. Hrycaj, Deneen M. Wellik

**Affiliations:** 1 Department of Internal Medicine, University of Michigan, Ann Arbor, Michigan, 48109-2200, USA; 2 Department of Cell and Developmental Biology, University of Michigan, Ann Arbor, Michigan, 48109-2200, USA

**Keywords:** homeobox, AP patterning, hox function, regionalization, deregionalized

## Abstract

*Hox* proteins are a deeply conserved group of transcription factors originally defined for their critical roles in governing segmental identity along the antero-posterior (AP) axis in *Drosophila*. Over the last 30 years, numerous data generated in evolutionarily diverse taxa have clearly shown that changes in the expression patterns of these genes are closely associated with the regionalization of the AP axis, suggesting that *Hox* genes have played a critical role in the evolution of novel body plans within Bilateria. Despite this deep functional conservation and the importance of these genes in AP patterning, key questions remain regarding many aspects of *Hox* biology. In this commentary, we highlight recent reports that have provided novel insight into the origins of the mammalian *Hox* cluster, the role of *Hox* genes in the generation of a limbless body plan, and a novel putative mechanism in which *Hox* genes may encode specificity along the AP axis. Although the data discussed here offer a fresh perspective, it is clear that there is still much to learn about *Hox* biology and the roles it has played in the evolution of the Bilaterian body plan.

## Introduction

Hox proteins are a group of homeodomain-containing transcription factors that are renowned for their roles in patterning animal body plans and for their remarkably deep evolutionary conservation. Homeodomain proteins are defined by the presence of a highly conserved DNA-binding region known as the homeodomain and are encoded by Homeobox genes. In general, homeobox genes are a large family of similar genes and can be divided into 11 different gene classes in animals, and the *Hox* genes belong to the ANTP class ^1, 2^. This class of genes also includes the closely related ParaHox genes, NK genes, and various others. It has been suggested that the evolution and expansion of *Hox* genes have played a key role in the rapid diversification of the body plans of all Bilaterians. Thus, this group of genes has fascinated evolutionary biologists for decades and continues to be studied by many research groups today.

*Hox* genes were originally discovered in *Drosophila* and functional studies in the fly showed that these genes play a critical role in establishing segmental identity along the antero-posterior (AP) axis ^3^. Subsequent analyses have shown that the role of *Hox* genes in establishing AP axis identity is conserved in vertebrates ^4– 6^. These data were very exciting and confirmed that their function was conserved in evolutionarily distant taxa. Since their original discovery in the fly over 30 years ago, *Hox* genes have now been cloned and analyzed in a wide array of animal groups ranging from hydra to humans. Collectively, these studies have provided key insights into the evolutionary origins of *Hox* genes and have reinforced the important role these genes have played in the evolution of Bilaterian body plans.

In this review, we provide a commentary on the recent advances on the origin, functional conservation, and regulative properties of *Hox* genes. The purpose of this review is not to provide a comprehensive detailed survey of the literature to date but rather to highlight recent data that have both challenged traditional views and enhanced our understanding of *Hox* genes and evolution.

## Evolution of the Hox genes

None of the ANTP class of homeobox genes (including the *Hox* genes) is found outside of the metazoans ^2^. During the evolution of metazoans, the sponges diverged first, followed by cnidarians (jellyfish and corals), and both of these groups are more basal to the Bilaterians. Analysis of whole genome information from the demosponge *Amphimedon queenslandica* revealed the first conclusive evidence that sponges have several NK homeobox genes but do not have any definitive *Hox* or ParaHox genes ^7^. In contrast, definitive *Hox*-like genes have been identified in the Cnidarians; however, the expression patterns of these genes do not follow a clear AP pattern or show any correlation with the Bilaterian *Hox* code in determining axis specification ^8^. Phylogenetic analyses of ANTP class genes have shown that the *Hox* and ParaHox genes are more closely related to each other than they are to the NK subclass ^1, 7^. Therefore, the current collection of genomic and phylogenetic data support the hypothesis that the NK, *Hox*, and ParaHox genes arose prior to the emergence of Bilaterian animals. Furthermore, it has been proposed that all three gene subclasses are derived from a hypothetical ancestral ANTP class gene that underwent extensive tandem gene duplications that ultimately created the three distinct gene clusters ^1^. Interestingly, each of these three gene clusters has been conserved to different extents in various evolutionary lineages within Bilateria ^1, 8^. For example, vertebrates have tightly linked *Hox* and ParaHox clusters and disrupted NK clusters, whereas dipterans (including *Drosophila*) exhibit a disrupted *Hox* cluster but have retained a tight NK cluster ^1^. Despite these differences, the birth and diversification of ANTP class genes have been instrumental in the evolution of the Bilaterian body plan and have contributed to the subsequent radiation of these animal taxa into nearly every ecological niche on earth.

Typically, invertebrates possess a single *Hox* cluster, whereas vertebrates possess multiple clusters that differ among different taxa ^9^. For example, mammalian genomes have four *Hox* clusters whereas teleost fishes have up to eight *Hox* clusters ^9– 11^. Although *Hox* genes and clusters are relatively well characterized in most vertebrates, the evolution of these genes within this group remains largely obscure because of the incompletely resolved phylogenetic history of these genes ^12^. In particular, the evolutionary origins of the *Hox*-bearing chromosomes in mammals remain highly controversial. The classic view is that the four clusters of *Hox* genes in humans originated through two rounds of whole genome duplications ^13– 15^. However, over the past few years, with the rapidly increasing availability of high-quality whole genome sequence data from a variety of animal species, the evolutionary history and organization of mammalian *Hox* genes have been subjected to rigorous scrutiny ^12, 16– 20^. Analyses of these emerging genomic datasets with advanced phylogenetic techniques have generated data that are inconsistent with the whole genome duplication hypothesis and instead favor the hypothesis that the configuration of *Hox*-bearing chromosomes in mammals may have resulted from small-scale events early in vertebrate evolution that include segmental duplications, independent gene duplication, and translocations ^12^. Such advanced phylogenetic techniques will continue to prove valuable and will provide more rigorous analyses of the evolution of the *Hox* genes as more high-quality whole genome sequence data from more basal metazoan taxa become available.

## Conservation of *Hox* function in antero-posterior patterning

The spatial and temporal expression patterns of *Hox* genes along the AP axis of flies reflect their position on the chromosome: genes at the most 3′ end are expressed earlier in development in more anterior parts of the embryo, and genes at the more 5′ position are expressed later in development in more posterior regions of the embryo ^9^. Studies in mice have shown the spatial and temporal expression patterns of these genes are also correlated with their position from 3′ to 5′ in each cluster, indicating that the spatial and temporal collinearity of the *Hox* genes is conserved in mammals ^4– 6, 21^. To date, *Hox* gene expression analyses in the vertebral column have been extended into several vertebrate taxa, including teleost fishes ^22^, squamates ^4, 23– 25^, and archosaurs ^4, 26, 27^. Comparative analyses of the *Hox* code in several amniote taxa provide strong evidence that the evolutionary differences in the axial skeleton correspond to changes in the expression domains of *Hox* genes ^26^. As more diverse taxa are sampled, the trend of deep conservation of the spatio-temporal expression and function of the *Hox* genes along the AP axis seems to be continually reinforced and underlies the critical roles that these genes have played in the evolution of the Bilaterian body plan.

There is an overwhelming amount of data that support that *Hox* genes are critical for patterning the axial skeleton in vertebrates and that changes in *Hox* gene expression have helped shape the evolution of novel body plans within Bilateria ^4, 5^. With these cumulative results, the origin of the snake-like body plan (as well as other snake-like squamates) with its “deregionalized” axial skeleton ^28, 29^ has been an intriguing evolutionary feature that has received considerable attention over the last decade with regard to *Hox* gene expression and function ^23, 25, 28– 30^. In limbed lizards, two distinct regional boundaries are observed in the axial skeleton: the cervical-thoracic and the thoracic-lumbar, both of which have been shown to correspond to sharp boundaries of differential *Hox* gene expression patterns ^23, 25, 28^. In contrast, it has been reported that the snake-like body plan lacks clear boundaries, resulting in a “deregionalized” axial skeleton with an increased number of vertebra and ribs and a reduction or loss of limbs and sternum ^28, 29^. Previous studies in mice have shown that the inactivation of all three genes in the *Hox10* paralogous group ( *Hoxa10*, *Hoxc10*, and *Hoxd10*) results in the transformation of the ribless lumbar vertebrae into a posterior extension of the thorax, as defined by the presence of ectopic ribs ^31, 32^. These and many other genetic studies demonstrate that the activity of the genes in the *Hox10* paralogous group controls key processes in somatic patterning that lead to the inhibition of rib development. However, expression analyses in snake embryos have shown that both *Hoxa10* and *Hoxc10* are expressed in rib-bearing regions of the axial skeleton, suggesting the possibility that snake *Hoxa10* and *Hoxc10* genes have lost the ability to suppress rib-bearing vertebrae ^25, 30^. Generation of transgenic mice that ectopically express snake *Hoxa10* showed that this paralog is able to efficiently block rib formation in mice, indicating that rib-repressing properties are still present in the snake protein ^33^. Instead, a polymorphism was identified in a Hox/Pax-responsive enhancer that is involved in Hox-mediated regulation of rib formation, which results in this enhancer being unable to respond to Hox10 proteins ^33^. In addition, this polymorphism was also found in other animals with extended rib cages. These data indicate that the evolution of this Hox/Pax enhancer has played a critical role in the evolution of axial skeletons by modulating responses to either rib-suppressing or rib-promoting *Hox* genes.

A recent report that more closely analyzed the morphological differences of snake vertebrae has challenged the traditional view that the anterior axial skeleton of snakes is, in fact, “deregionalized” ^28^. Using a statistical geometric morphometric analysis on the vertebral morphology, Head and Polly ^28^ concluded that there was no consistent difference in the shape variance between limbed and snake-like squamates and that three to four distinct vertebral regions, including the cervical, thoracic, and lumbar regions, could be identified in all taxa irrespectively of the presence or absence of limbs. In other words, snake-like body plans do indeed have regionalized precloacal axial skeletons; the differences in the morphologies of the vertebrae are just more subtle as compared with limbed reptiles. In addition, the authors asserted that the newly identified morphological boundaries of the snake vertebral columns correspond to similar mapped expression boundaries of *Hox* paralogs in snakes, suggesting that the AP axis of these animals is governed by a normally functioning *Hox* code.

From an evolutionary perspective, the “deregionalization” of the snake axial skeleton made the assumption that this body plan evolved from an ancestor that exhibited a regionalized AP vertebral axis. The new data reported in Head and Polly challenge this assumption and instead suggest that the regionalized axial skeletons of limbed reptiles and other derived vertebrate taxa are descended from an axial plan that displayed very little regionalization in the first place ^28^. Indeed, this hypothesis is supported by acquired fossil evidence from Paleozoic amniotes, including extinct stem members of Reptilia and Mammalia, that shows that these animals exhibited “deregionalized” axial skeletons with very subtle changes in their primaxial morphology ^28^. These data support a model wherein regionalized vertebral columns (including the ones in snakes) are a derived feature that has arisen through modifications of a more “deregionalized” ancestral body plan. In this case, the evolution of the snake-like body plan is not an exception but rather just another example along the continuum of *Hox* function in sculpting derived body plans in the diverse Bilaterian taxa.

In addition to their highly conserved roles in AP patterning, numerous studies have indicated that *Hox* genes have been co-opted for novel functions in the development of many organ systems. For example, previous studies have shown that the expression patterns of the *Hox* gene *Ultrabithorax* ( *Ubx*) are associated with the differential enlargement of particular hind-limb segments in different insect species ^34, 35^. In a similar fashion, the *Hox* gene *Abdominal-B* is required for the formation of the lantern organ on the posterior abdomen in the firefly ^36^. Although studies in insects are informative, our most comprehensive understanding of co-opted *Hox* gene functions comes from studies in mice. These studies have identified several important roles for *Hox* genes in the development of organs that correspond to their expression along the AP axis. Some of the many examples include the following: the *Hox3* genes in the development of the thymus, thyroid, and parathyroid ^37, 38^; *Hox5* genes in lung development ^39, 40^; *Hox6* genes in pancreas development ^41^; *Hox9*, *Hox10*, and *Hox11* genes in the reproductive tract ^42– 46^; *Hox10* and *Hox11* genes in kidney development ^47– 49^; and *Hoxb13* gene for prostate development ^50^. Although *Hox* genes have been shown to play important roles in many aspects of organogenesis, it has been difficult to place these highly conserved transcription factors into established regulatory networks. This represents an important gap in our understanding of *Hox* biology that needs to be addressed in much greater detail.

It has been well established that the diversity along the AP axis of animals results from the differential expression of *Hox* genes, which in turn regulate different sets of target genes that govern the formation of anatomical regions that have specific features ^51^. However, how *Hox* genes encode this specificity has been a long-debated question. All Hox proteins have similar DNA-binding domains (the homeodomain) and they all bind similar DNA sequences with high affinity ^51– 56^. One well-established means by which *Hox* genes achieve specificity *in vivo* is to bind DNA co-operatively with other DNA-binding co-factors ^55, 57^. To date, the three amino acid loop extension (TALE)-homeodomain genes, which include the PBC/PBX and MEIS classes of homeodomain proteins, are the best described co-factors; however, it is clear that others exist ^55, 57– 61^. The PBC/PBX class comprises fly Extradenticle (Exd) and vertebrate Pbx homeoproteins, whereas the MEIS class includes fly Homothorax (Hth) and vertebrate Meis and Prep homeoproteins ^57^. In addition to the presence or absence of co-factors, a recent report has significantly contributed to additional understanding of how *Hox* genes encode specificity in *Drosophila* ^54^. Briefly, these researchers identified clusters of low-affinity binding sites in enhancers of the *shavenbaby* ( *svb*) gene that specifically confer binding of an Ubx-Exd complex. Mutation of these sites into high-affinity sites enabled the enhancer to respond to other *Hox* genes ( *Scr*), suggesting that the native low-affinity Ubx-Exd binding sites confer specificity for Ubx-Exd dimers over other Hox proteins and probably over other homeodomain proteins as well. Interestingly, although the individual Ubx binding sites were not conserved in another fly species ( *Drosophila virilis*), clusters of other low-affinity binding sites were identified and found to be required for enhancer function, suggesting that this mechanism may be an evolutionarily conserved strategy used by *svb* enhancers. Determining whether similar mechanisms convey *Hox* specificity in more derived Bilaterian species will be particularly informative and will provide insight into whether this mechanism is a highly conserved feature.

## Future directions

The remarkably deep conservation of *Hox* gene organization and function and their profound impact on the evolution of metazoan body plans continue to fascinate evolutionary and developmental biologists today. As a result, *Hox* genes continue to be investigated by a large number of research groups. The focus of these studies encompasses many different aspects of *Hox* biology, including *Hox* gene regulation, identification of downstream targets, and uncovering novel functions for these proteins. In addition, *Hox* genes have been associated with a number of human diseases ^62^ and this in turn supports an increased need to understand the potential role(s) of these genes in the onset and progression of disease. Finally, functional roles of *Hox* genes have also been identified during postnatal development ^63– 65^, and there is increasing interest in understanding the roles that these genes play in the formation of post-embryonically derived structures and the maintenance of organ systems. There remains much more to learn about *Hox* gene biology and thus it is certain that these genes will continue to fascinate investigators for decades to come.
